# Clinical outcome analysis in surgical patients enrolled in a Second Opinion Program in spine surgery

**DOI:** 10.31744/einstein_journal/2022AO5791

**Published:** 2022-03-22

**Authors:** Rebeca Barqueiro de Oliveira, Isadora Orlando de Oliveira, Eliane Antonioli, Mario Lenza, Mario Ferretti

**Affiliations:** 1 Faculdade Israelita de Ciências da Saúde Albert Einstein Hospital Israelita Albert Einstein São Paulo SP Brazil Faculdade Israelita de Ciências da Saúde Albert Einstein, Hospital Israelita Albert Einstein, São Paulo, SP, Brazil.; 2 Hospital Israelita Albert Einstein São Paulo SP Brazil Hospital Israelita Albert Einstein, São Paulo, SP, Brazil.

**Keywords:** Spinal diseases/surgery, Arthrodesis, Pain management, Low back pain, Anxiety, Depression, Treatment outcome, Referral and consultation, Quality of life

## Abstract

**Objective:**

To analyze pain, functional capacity, quality of life, anxiety and depression outcomes in patients undergoing lumbar spine surgery following use of the Second Opinion Program, and to present disagreements regarding diagnoses and therapeutic indications between the first and second opinions.

**Methods:**

A prospective, observational cohort study with 100 patients enrolled in the Second Opinion Program who underwent lumbar spine surgery. Questionnaires addressing pain intensity, level of disability, quality of life, anxiety and depression were applied prior to and within 1, 3, 6 and 12 months of surgery. Descriptive and comparative statistical analyses were performed. The following clinical outcomes were analyzed: pain intensity, level of disability, quality of life, anxiety, and depression.

**Results:**

In this sample, 88% and 12% out of 100 patients were submitted to lumbar decompression and arthrodesis, respectively. Patients reported improvements in function, pain intensity, and quality of life factors following surgery and were able to attain the minimal clinically important difference relative to the preoperative period. Agreement between the first and second opinions was observed in 44% of diagnoses, and in 27% of therapeutic indications.

**Conclusion:**

Patients had favorable postoperative outcomes regarding pain, disability, and quality of life. These findings and the high rates of diagnostic and therapeutic indication disagreements corroborate the need of a second opinion in cases of spine disease with surgical indications.

## INTRODUCTION

Degenerative spine diseases affect a large proportion of the active population. The chronic nature of these conditions has negative impacts on patient quality of life and functional capacity, leading to limitation in activities of daily living, absenteeism, and high health care costs.^(1)^

An estimated 80% of affected individuals report low back pain within 12 months of the first back pain episode and tend to seek treatment alternatives more frequently.^(2)^ In some cases, the condition may progress to sciatica due to involvement of nerve roots, resulting in radiating pain to the lower limbs.^(3)^

Surgical management of spine diseases is being increasingly indicated,^(4)^ particularly in cases with severe sciatica or neurologic changes.^(3)^ According to recent national and international clinical guidelines, conservative management consisting of physical therapy, acupuncture and other non-invasive methods should be the primary recommendation for patients with chief complaint of low back pain and sciatica pain, except in cases with red flags.^(5)^

Multidisciplinary follow-up and use of validated questionnaires are vital to measure patient experience and treatment progression. Second Opinion Programs (SOP) aimed to improve patient quality of life, and reduce spine disease treatment costs are also important.^(6)^

In 2011, a SOP program named *Projeto Coluna* (Spine Project) was developed at *Hospital Israelita Albert Einstein* (HIAE) to refine the approach and management of patients suffering from degenerative spine diseases. This initiative is a collaboration between HIAE and Brazilian health insurance companies and offers an alternative for patients seeking a second medical opinion.^(6)^

The high number of surgical indications, the scarcity of studies supporting treatment effectiveness, the need of post-procedure follow-up and the rising number of second opinion visits in surgical cases justify novel studies investigating the role of SOP in the health system.^(6)^

This study set out to examine the clinical outcomes of patients enrolled at SOP who underwent spine surgery, and to identify the rate of disagreements regarding diagnoses and therapeutic indications between the first and second opinions in spine surgery.

## OBJECTIVE

To analyze pain, functional capacity, quality of life, anxiety and depression outcomes in patients undergoing to lumbar spine surgery following use of the Second Opinion Program, and to present disagreements regarding diagnoses and therapeutic indications between the first and second opinions.

## METHODS

This is a prospective, observational cohort study comprising 100 patients who agreed to the treatment proposed by the HIAE SOP. Inclusion criteria were individuals aged over 18 years with initial indication for surgical treatment due to degenerative spine conditions (such as intervertrebral disc disease, spondylolisthesis and spinal stenosis), no contraindications for general anesthesia or physical therapy, and good command of the Portuguese language.

Patients with spinal fractures, scoliosis greater than 20^o^, congenital deformities, tumors, confirmed or suspected pregnancy, previous spine surgery, or follow-up-related constraints (inability to read or complete questionnaires) were excluded.

The SOP of HIAE was launched in 2011 and introduced a much needed alternative prior to therapeutic decision making. For patients with indications for lumbar spine surgery according to credentialled physicians, the health insurance companies offer a second opinion visit at HIAE. These patients are then seen by a SOP rehabilitation physician or orthopedic surgeon. Visits consist of history taking, physical examination and analysis of previous test results. Each physician described the presumptive diagnoses, and ordered other tests as needed.

In the SOP, conservative or surgical management may be indicated. In cases with indications for conservative treatment, patients can choose between treatment at HIAE or going back to their primary physician. In cases with surgical indication, patients are referred for the SOP surgical team using randomization criteria.

Surgical approaches are selected during group discussions with orthopedic surgeons, neurosurgeons, and members of the multidisciplinary team (Spine Board), according to HIAE protocols and guidelines. These meetings are held weekly. Spinal decompression or arthrodesis at no more than two spinal levels may be indicated by the SOP team.

Following spine Board decisions, treatment indications are communicated and discussed with the patient, who will then decide whether to go forward with the proposed procedure or seek external medical services.

In this study, a total of 100 surgical patients were recruited by the SOP, between February 6, 2017 and November 29, 2018, and prospectively followed for 12 months after surgery.

### Data collection

Patients who accepted the SOP proposal were immediately invited to participate by the team’s nurse. Those who accepted to participate signed an Informed Consent Form. Data were collected using questionnaires. The following pieces of data were collected: sociodemographic profile, diagnosis and treatment indication given by external and SOP physicians, severity of lumbar pain, functional disability, quality of life, anxiety, and depression. Questionnaires assessing clinical outcomes were applied in the preoperative period and within 1, 3, 6 and 12 months of surgery.

The Brazilian version of the Oswestry Disability Index (ODI) was used to investigate spine-related functional disability. This questionnaire comprises ten questions addressing daily living domains,^(7)^ with six response alternatives rated on a zero to five scale. The final score corresponds to the summed response score multiplied by two. Final scores range from zero (no disability) to 100% (maximum disability).^(8)^

Pain intensity was assessed using the Visual Numerical Scale (VNS). This scale ranges from zero (no pain) to ten (maximum pain) and only one whole number must be selected.^(5,9)^

Anxiety and depression were assessed using the validated Portuguese version of the Hospital Anxiety and Depression Scale (HADS). This questionnaire comprises seven questions addressing anxiety and seven questions addressing depression (HADS-A and HADS-D, respectively). Maximum scores per domain is 21 points (summed score of 7 items rated 0 to 3). Scores higher than eight are a red flag for psychological symptoms.^(10)^

The EuroQoL-5D-3L (EQ-5D-3L) was used to measure quality of life. This questionnaire comprises five domains (mobility, self-care, activities of daily living, pain and discomfort, anxiety and depression) and three response alternatives (no problems, moderate problems or extreme problems). The closer to one, the better the quality of life; the closer to zero the worse the quality of life.^(11)^

The following strategies were applied to minimize losses to follow-up: briefing about the importance of questionnaires and adherence to the study, scheduled (date and time) follow-up questionnaire application according to participant’s preference, matching follow-up questionnaire and medical visits to minimize the need to travel to the hospital, and regular update of telephone numbers and e-mail addresses.

### Sample size calculation

This study is part of a cohort comprising 100 patients undergoing surgical treatment and 90 patients undergoing conservative treatment following use of the SOP. Initial sample size estimation was based on van der Roer et al.^(12)^ In that study, the mean pretreatment EuroQoL score achieved by chronic patients was 0.70 (standard deviation, 0.19). Assuming a correlation of 0.5 and a minimal clinically important difference of 0.07 between measurements made at baseline and after 3 months, a sample size of 100 patients was deemed ideal for the surgical intervention group (assuming 55% of chronic patients would use the second opinion service).

Calculations were made using STATA, version 10.0. Statistical power was set at 90% and the level of significance at 5%.

### Statistical analysis

Demographic data were expressed as mean, median and standard deviation. Outcome data were expressed as mean scores.

The minimal clinically important difference (MCID) according to disability (>12%), pain (>2 points) and quality of life (>0.03 points) questionnaires was also investigated.^(13)^

Quantitative data and parameters were analyzed with the assistance of the statistics consulting services of *Instituto Israelita de Ensino e Pesquisa* (IIEP).

Patients were duly informed of their rights, follow-up periods, and the possibility of withdrawing their participation at any time after signing the Informed Consent Form.

This study was approved by the Research Ethics Committee of *Hospital Israelita Albert Einstein*, opinion # 1.804.215, CAAE: 59736016.0.0000.0071.

## RESULTS

**Figure 1:**
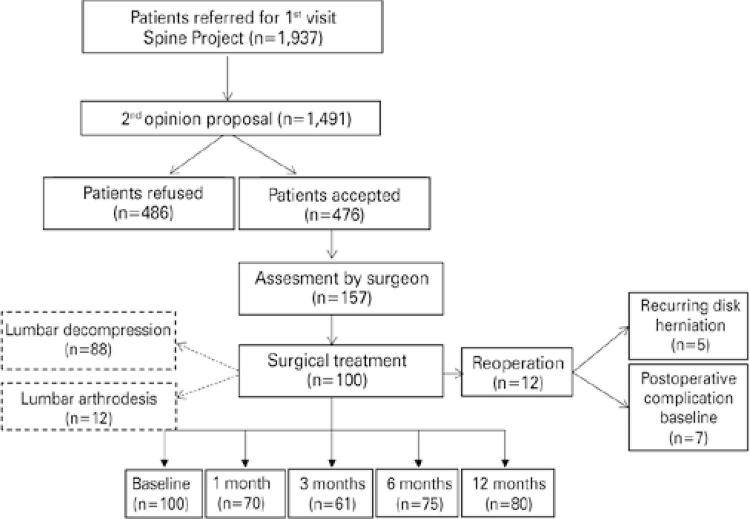
Flowchart of patients enrolled in the Second Opinion Program

Over the course of the experimental period, 1,937 patients were referred to the SOP by health insurance companies. Of these, 1,491 were eligible, 476 accepted the SOP proposal, and 157 were referred for surgical assessment. A total of 100 patients agreed to participate. Lumbar decompression and lumbar arthrodesis were indicated in 88% and 12% cases, respectively. Eleven patients in the lumbar decompression group had to be reoperated due to recurrent disc herniation, infection, or hematomas. Only one patient in the lumbar arthrodesis group had to be reoperated due to infection (Figure 1).

Postoperative follow-up was carried out within 1, 3, 6 and 12 months of surgery. Some patients did not return for follow-up questionnaire application. Within 1 month of surgery, 29 cases were lost to follow-up and one patient was hospitalized. In the third and sixth months, 39 and 25 cases were lost to follow-up, respectively. Within 12 months of surgery, 8 cases were lost to follow-up, one patient died, and one patient was hospitalized.

Demographic data and characteristics of patients included in the study are shown in table 1.

**Table 1:** Descriptive data of patients included in the study

Characteristics	
Age, years, SD	46.0 (15.2)
Male sex	51
Weight, kg	82.2±17.2
Height, m	1.70±0.10
BMI, kg/m^2^	28.3±4.9
Smoking, %	12
Leve of education, n (%)	
Junior school	10 (10.0)
High school	21 (21.0)
Technical	1 (1.0)
Further education	68 (68.0)
Marital status, n (%)	
Single	16 (16.0)
Married	77 (77.0)
2Divorced	6 (6.0)
Widow (er)	1 (1.0)
Radiating pain to lower limbs	89
Reoperation	5
ODI (0-100%)	46.98±18.50
VNS (0-10)	7.17±2.45
HADS-A (0-21)	8.24±4.64
HADS-D (0-21)	6.13±4.56
EQ-5D-3L (-1-1)	0.255±0.378

BMI: body mass index; ODI: Oswestry Disability Index; VNS: Visual Numerical Scale; HADS-A: Hospital Anxiety and Depression Scale- anxiety; HADS-D: Hospital Anxiety and Depression Scale-depression; EQ-5D-3L: EuroQol-5D-3L.

Scores obtained over the course of postoperative follow-up are shown in table 2. Spine-related disability scores (ODI) dropped considerably within 1 month of surgery and remained unchanged thereafter, with an 83% drop in ODI, 12 months after surgery.

**Table 2:** Descriptive measures for scores applied to patients with chronic low back pain (n=100) before and after surgical treatment

Questionnaires	Assessment				

	Baseline	1 month	3 months	6 months	12 months
VNS					
Mean±SD	7.4±2.3	2.3±2.2	2.8±2.7	1.8±2.4	2.2±2.3
Min-max	0-10	0-10	0-10	0-10	0-8
Total	100	70	61	75	80
ODI					
Mean±SD	47.2±18.3	16.8±13.4	16.4±17.1	10.0±11.9	7.8±10.4
Min-max	6-96	0-60	0-86	0-48	0-56
Total	100	70	61	75	80
EQ-5D-3L					
Mean±SD	0.260±0.380	0.670±0.264	0.749±0.263	0.787±0.242	0.834±0.214
Min-max	-0.594-0.883	-0.113-1.000	-0.181-1,000	-0.135-1.000	-0.016-1.000
Total	100	70	61	75	80
HADS-D					
Mean±SD	6.3±4.6	3.5±3.7	3.5±4.0	2.7±3.8	1.7±3.5
Min-max	0-19	0-14	0-18	0-20	0-21
Total	100	70	61	75	80
HADS-A					
Mean±SD	8.3±4.5	5.4±4.5	4.6±3.8	3.6±3.4	2.9±3.7
Min-max	0-20	0-18	0-17	0-19	0-21
Total	100	70	61	75	80

VNS: Visual Numerical Scale; SD: standard deviation; ODI: Oswestry Disability Index; EQ-5D-3L: EuroQoL-5D-3L; HADS-A: Hospital Anxiety and Depression Scale; HADS-D: Hospital Anxiety and Depression Scale; Min-max: Minimum-maximum.

Patients reported moderate pain intensity prior to surgery (mean VNS pain score, 7). Pains scores dropped significantly (mean VNS pain score, 2) within 1 month of surgery, with approximately 70% reduction in pain intensity within 12 months. Pain scores did not differ significantly across remaining follow-up assessments.

EQ-5D-3L scores increased over the course of follow-up relative to baseline. EQ-5D-3L were significantly higher at 12 relative months relative to one month after surgery.

Score variations across different follow-up time points are shown in table 3. Assuming MCIDs of 12%, 2 points and 0.03 points for disability, pain, and quality of life respectively, data presented indicate patients improved after surgery. Patients in both groups attained higher MCID values in the first month after surgery. These values remained unchanged within 12 months of surgery. The expected MCID value for HADS questionnaire has not been described in the literature yet. Hence, these values were not presented.

**Table 3:** Minimum clinically important difference attained by surgical patients over the course of follow-up

Questionnaires	Follow-up			

	MCID 1 month	MCID 3 months	MCID 6 months	MCID 12 months
ODI (0-100%)				
Decompression	30.00	29.55	36.04	39.30
Arthrodesis	28.58	42.73	45.76	39.89
VNS (0-10)				
Decompression	4.65	4.39	5.29	5.10
Arthrodesis	5.79	6.17	7.17	5.28
EQ-5D-3L (-1–1)				
Decompression	0.432	0.479	0.509	0.581
Arthrodesis	0.337	0.605	0.681	0.534

Decompression: n=88; arthrodesis: n=12.

MCID: minimum clinically important difference: ODI: Oswestry Disability Index; VNS: Visual Numerical Scale: EQ-5D-3L: EuroQol-5D-3L.

The analysis revealed disagreement on diagnoses and therapeutic indications between the first and second opinions in 56% and 73% of cases, respectively. External physicians recommended high-complexity interventions (lumbar arthrodesis) in 47% of cases, with only 31% patients allocated to lumbar decompression. In contrast, less complex interventions (lumbar decompression) were indicated in most cases (88%) seen by the SOP team. According to the first opinion, ten patients would require more than one procedure. Hence, the number of procedures totaled up 110 (Table 4).

**Table 4:** Disagreements in treatment indications in patients included in the study

Procedures	First opinon	Second opinion
Lumbar decompression	31	88
Lumbar arthrodesis, 1 level	20	7
Lumbar arthrodesis, 2 levels	20	5
Lumbar arthrodesis, 3 levels	5	0
Lumbar arthrodesis, 4 levels	1	0
Lumbar arthrodesis, 5 levels	1	0
Lumbar infiltration, 1 level	12	0
Lumbar infiltration, 2 levels	6	0
Lumbar infiltration, 3 levels	3	0
Lumbar infiltration, 4 levels	1	0
Lumbar infiltration, 5 levels	1	0
Lumbar infiltration, 6 levels	1	0
Rhizotomy	2	0
Discography	6	0
Total	110	100

## DISCUSSION

In this study, patients undergoing surgical treatment after enrollment in the SOP achieved better functional capacity, pain, and quality of life outcomes. This study also revealed significant disagreements regarding diagnoses and therapeutic indications between the first and second medical opinion among physicians.

According to Del Castillo et al., surgical patient follow-up using specific questionnaires is extremely important for appropriate case management.^(14)^ In this study, follow-up assessment and validated questionnaires were used to measure improvements in the functional capacity, pain intensity, and quality of life.

Low back pain is a heterogeneous condition. Therefore, affected patients are not well-characterized. Higher prevalence has been reported among men in some studies,^(15,16)^ whereas in others the condition was more prevalent among women, often due to inappropriate posture, household chores, and smaller muscle mass.^(17-19)^ In this study, pain perception was not associated with sex (*i.e.,* low back pain affected men and women alike).

In recent studies, patients undergoing spine surgery were aged over 50 years on average.^(18,19)^ This is thought to reflect bone, ligament, and muscle changes, loss of flexibility and adoption of inappropriate postures over time, along with age-related comorbidities and degenerative diseases, such as arthritis.^(20)^ Patients in this sample were comparatively younger (mean age, 46 years). It has been suggested that the daily routine of working age individuals living in large cities may be related to early development of chronic low back pain. This may be explained by changes in intervertebral disk pH and nutrition induced by factors, such as longer working hours, long commuting, and greater exposure to tobacco chemicals.^(19)^ In this population, leisure time, preventive health care, and search for quality of life, are often not prioritized.^(19)^

Psychosocial compromise may interfere with assessment of outcomes, such as functional capacity and quality of life. Biopsychosocial factors are thought to have significant impacts on postoperative outcomes in patients submitted to spine surgery.^(21)^

Depression and anxiety are the most widely reported psychological factors among patients scheduled for spine surgery and are associated with poorer outcomes.^(22)^ Pain catastrophizing may also have negative impacts on postoperative pain intensity and functional capacity levels. Hence, the significance of psychologic screening and risk factor identification in patients with persistent pain.^(23)^

Some patients who did not report pain had functional impairments and low quality of life perception. These findings suggested, even in the absence of pain, these patients faced difficulties in activities of daily living, with direct impacts on their quality of life. However, lumbar spine surgery may have positive impacts on pain intensity, functional capacity, and quality of life even in patients with moderate biopsychosocial risk.^(22)^

Improvements in psychosocial and quality of life outcomes in this study emphasized the relevance of appropriate follow-up and care of patients with chronic low back pain. Findings presented are congruent with the current literature regarding the value of preoperative screening via application of validated questionnaires, and identification of factors with potential impacts on patient outcomes for timely intervention.^(23)^

Radiating pain to lower limbs in response to nerve root compression is another common clinical condition with surgical indication in patients suffering from degenerative spine diseases.^(2)^ In one study, 71% of patients undergoing spine surgery reported radiating pain to lower limbs prior to surgery.^(24)^ Likewise, 89% of patients in this study had preoperative sciatica.

As to therapeutic indication disagreements, a prospective study with 437 patients with cervical or low back pain revealed differences between the first and second medical opinions in 41.9% of cases. In that sample, only 6% of patients were given similar therapeutic advice in the first and second visits.^(25)^ In this study, similar therapeutic recommendation were given in only 27% of cases. These findings emphasize the significance of a second medical opinion for enhanced assertiveness, since therapeutic alternatives are discussed among several specialists, according to institutional guidelines.

With regard to surgical interventions indicated for degenerative spine conditions, lumbar decompression is a less invasive and cheaper procedure, with shorter length of hospital stay, and lower complication rates.^(26)^ In this study, this indication prevailed in approximately 90% of cases seen by the SOP team and translated into clinical improvement. Reoperation rates of 6% have been reported in patients submitted to open or endoscopic lumbar decompression due to recurrent disk herniation.^(27)^ Similar reoperation rates (5%) have been detected in this sample.

Lumbar arthrodesis is a more complex procedure relative to lumbar decompression, with higher complication rates in more than 12% of cases.^(28)^

This study has some limitations, such as poor patient adherence to follow-up questionnaires, outcome data losses due to remote follow-up after the first month, short postoperative follow-up, and lack of follow-up of patients who refused to use the SOP.

Future studies investigating SOP and management of cases are warranted. Findings of such studies may be help refine follow-up strategies, contribute to clinical practice, reduce over-indication of surgical treatment, encourage multidisciplinary approaches and adjust the allocation of resources available by health insurance companies, hospital, patients and respective families.

## CONCLUSION

Participants enrolled in the Second Opinion Program achieved significant improvements in functional capacity, low back pain intensity, quality of life, and psychosocial factors following surgery, and were able to attain the minimum clinically important difference across all follow-up time points. Findings of this study emphasized the significance of the Second Opinion Program, given the high rates of disagreements regarding diagnoses and therapeutic indications in patients with chronic low back pain.
